# A System of Diet and Dietetics

**Published:** 1909-09

**Authors:** 


**A System of Diet and Dietetics.** Edited by G. A. SUTHERLAND, M.D., F.R.C.P. Pp. xiii, 893. London: Oxford Medical Publications. 1908.

This is a work of great and general interest on a most difficult subject, in which scientific opinion, as well as popular belief, has not attained to certainty. As the editor remarks, "until our knowledge of physiology is more perfect than at present, the scientific basis of dietetics must be an unstable one." The volume is composed of articles by different writers, each of whom has special knowledge of the subject which he undertakes. Complete agreement among them is not to be expected. Moreover, each article represents the views and experience of the writer, rather than a summary of the teaching of different authorities, though many of the chief theories are discussed. The distinguished writers whom the editor has enlisted have given us a collection of papers of remarkable interest and value.

After an introduction by Sir T. Lauder Brunton, Dr. Harry Campbell sketches the evolution of man's diet in the past, and shows the practical value of such a survey. Thus he points out that there is no historical basis for vegetarian theories. The enormous increase of sugar and soft carbohydrate food stuffs in modern times seems to have outstripped the evolution of the digestive organs, and may explain the prevalence of constipation, dyspepsia, and several other disorders in this "age of pap foods."

Our present knowledge of the physiology of digestion, and the results of experimental work on diet, is ably summarised by Dr. E. I. Spriggs. The various tables of heat values of foods and of the dietaries in actual use will be found to be of the greatest value for daily reference, and are given in much detail and clearness. A very readable account of the Chittenden controversy as to proteids completes his paper, though some of Chittenden's later arguments seem to be omitted. The article on "Dietetic Cures" is, perhaps, hardly detailed enough to be useful. One of the worst examples is the account of Weir Mitchell's diet, which is dismissed in nine lines. The writer, however, makes amends by supplying elaborate tables of the analyses of patent foods and their nutritive values.

One of the most interesting discussions is that on feeding patients during typhoid fever, by Dr. Claud Ker, who advocates a cautious and conservative method, but gives a critical review of the chief plans which have been put forward.

The article on "Tuberculosis" has been entrusted to Drs. Bardswell and Chapman, who not only describe the theoretically-perfect diet, but supplement it with most useful details as to how the best available diet can be selected and bought by the poor. These suggestions should be in the hands not only of medical men, but also of all lay committees and health visitors everywhere. Ordinary hospital diets might be much improved by the help of these tables.

Dr. G. A. Luff's paper on "Diet in Gout and Rheumatism" contains much original thought and practical sense. It may be noted that he shows little favour to "purin-free" diets. The chapter on "Obesity," by Dr. Cautley, is one of the most complete accounts of treatment which we have met with. Dr. H. P. Hawkins takes the important section on diseases of the stomach and intestines, and gives an eminently useful and readable article. We should have liked a fuller account of the Lenhartz treatment for gastric ulcer, and fewer German diet sheets with unfamiliar articles of food. The editor contributes two long chapters on diet in infancy and childhood, and there is probably no better authority on these subjects anywhere. His papers will be often consulted to good purpose. The work is admirably printed in good type, and appears to contain very few errata.

